# Assessment of risk factors for suicidal behavior: results from the Tehran University of Medical Sciences Employees' Cohort study

**DOI:** 10.3389/fpubh.2023.1180250

**Published:** 2023-08-22

**Authors:** Zahed Rezaei, Samira Mohammadi, Abbas Aghaei, Hamidreza Pouragha, Arman Latifi, Nastaran Keshavarz-Mohammadi

**Affiliations:** ^1^Social Determinants of Health Research Center, Gonabad University of Medical Sciences, Gonabad, Iran; ^2^Asadabad School of Medical Sciences, Asadabad, Iran; ^3^Health Metrics Research Centre, Iranian Institute for Health Sciences Research, ACECR, Tehran, Iran; ^4^Social Determinants of Health Research Center, Research Institute for Health Development, Kurdistan University of Medical Sciences, Sanandaj, Iran; ^5^Center for Research on Occupational Disease, Tehran University of Medical Sciences, Tehran, Iran; ^6^Department of Environmental Engineering, Mehralborz University (MAU), Tehran, Iran; ^7^Department of Public Health, School of Public Health, Research Center for Evidence-Based Health Management, Maragheh University of Medical Sciences, Maragheh, Iran; ^8^Department of Public Health, School of Health and Safety, Shahid Beheshti University of Medical Sciences, Tehran, Iran

**Keywords:** suicide, suicidal behavioral, employed in the medical area, demoralization, suicide ideation and behavior

## Abstract

**Introduction:**

Suicide is a major issue of concern for public health. It is estimated that suicide accounts for 700,000 deaths every year. A personal history of one or more suicide attempts is the most important determinant of suicide among the general population. This study aimed to assess the major risk factors associated with suicidal behaviors among Iranian employees in a medical setting.

**Methods:**

In this study, 3,913 employees of Tehran University of Medical Sciences who participated in the employees' cohort study conducted by the university were recruited. Suicidal behaviors (SBs) and their associated risk factors were evaluated using the World Mental Health Composite International Diagnostic Interview (CIDI) Version 3.0. Univariate and multivariate logistic regressions were performed to identify the determinants of SBs among the participants, and crude and adjusted odds ratios (ORs) with corresponding 95% confidence intervals (95% CIs) were calculated.

**Results:**

Overall, 49.6% of respondents (*n* = 1,939) reported that they were tired of life and thinking about death. The lifetime prevalence rate of suicidal ideation (SI) was 8.1% (*n* = 317), that of suicide planning (SP) was 7.3% (*n* = 287), and that of suicide attempts (SA) was 3.1% (*n* = 122). Being female (OR: 1.87, CI: 1.64–2.12), being divorced (OR: 3.13, CI: 1.88–5.22), having a low level of education (OR: 1.57, CI: 1.15–2.14), and working in clinical and medical services (OR: 1.25, CI: 1.09–1.43) were associated with being tired of life and thinking about death. These factors were also associated with SI, SP, and SA.

**Discussion:**

These findings highlight the need to prioritize mental health for suicide prevention, especially for high-risk groups, in workplace mental health promotion programs and policies.

## Introduction

Suicide is a major issue of concern for public health; globally, it is considered to be among the 20 most common causes of death. Every year, ~700,000 people die by suicide (1), although these deaths are preventable. The majority of suicide cases (~79%) are reported to occur in low- and middle-income countries, where financial and professional resources to help those in need are limited (2). Suicide is not an independent event; rather, it is a process that begins with suicidal ideation (SI); continues with disappointment, momentary SI, and precise plans; and finally ends with a suicide attempt (SA) (3).

Some studies suggest that total rates of SA are up to four times higher than the rate fatal SA (4–9). A study that investigated the prevalence of SI, suicide plans, and attempts among 84,850 adults from 17 countries reported that 9.2% of adults had experienced lifelong SI, but only 3.1% reported lifelong suicide planning (SP), and 2.7% reported at least one SA (10). However, the study also reported that 60% of people attempt suicide in the first year after SI. Among people with SI, there was a 33% probability of ever having planned suicide and a 29% probability of ever having attempted suicide. In addition, among the individuals who had experienced both SI and SP, ~56% had attempted suicide, as compared to the 15.4% rate of suicide attempts among those who had experienced only SI (10). Therefore, it is clear that SI increases the risk of suicide, and having an SP increases this risk even more (11). Most fatal SAs happen after one or two previous SAs (8, 12). The risk of recurrence of SA is at its highest in the first 6 months after an episode (13), during which period the rate of recurrence can be up to 9% (14). Within 1–2 years after an episode, ~60% of individuals experience recurrence of SA (15). In comparison, the rate of fatal SA during 1–4 years of follow-up is ~3% (16).

Studies have identified several potential determinants of or influencing factors for suicide and fatal SA, including social, biological, genetic, psychological, environmental, and local factors (17). For example, a history of mental illness, substance or alcohol abuse, chronic illness, emotional problems, violence, sudden major changes in a person's life (such as job loss or separation), lack of a source of income or insufficient income to make ends meet, and a history of suicide among immediate family members are the major determinants of suicide. A multiplicity of these factors in a particular individual will increase the chance of a fatal SA (10, 18–20). Suicide attempters are often diagnosed with other psychiatric disorders, including major depressive disorder, anxiety disorder, post-traumatic stress disorder, and substance abuse disorder. Most attempters also often report SI (21, 22). Fatal SAs are reportedly associated to a greater extent with severe clinical conditions (23, 24), as well as being influenced by gender and age (25). Statistics show that the rate of suicide is three to four times lower in males than in females (26, 27), but hospitalization due to SA is more common among females (28). However, the mortality rate of SA is three times higher in males than in females (26, 29). These differences are attributed to the higher prevalence of depression in females and the use of more lethal methods by men during SA (30).

Suicide is not easy to predict, as it is a complex multifactorial phenomenon that depends on the dynamics of various factors over time (4–9). In addition, the effects of risk factors may vary among different individuals based on their demographic characteristics, which are subject to change over time. For example, the effect of being single and unemployed and having a low income on the incidence of suicide is greater in men (31). Unpleasant psychosocial conditions in the workplace, known as social determinants of health (SDH) (32), such as low job control and high job demand (28), as well as reward imbalances, lead to poor mental health, which is a major predictor of suicide (33).

Mental health in the workplace is a crucial aspect of workplace health, as it influences both the health and the productivity of employees (33). According to a systematic review of suicide risk among healthcare workers, SI is a significant problem among these workers. The review found that healthcare workers have a higher risk of suicide than the general population. The authors also found that there is a lack of research on the topic of SI among healthcare workers (34). Hence, a key initial step for any effective and tailored workplace health promotion program is to identify the important mental health risk factors and high-risk groups within the environmental context of employees in terms of living and working. This article presents one component of a major workplace health cohort project conducted in Iran by the Tehran University of Medical Sciences (TUMS). This study aimed to assess the major risk factors associated with suicidal behavior (SB) among Iranian employees in medical settings.

## Materials and methods

### Study design and population

Data were extracted from the TUMS employees' cohort (TEC) study. Specifically, 3,913 employees who participated in the TEC study, which was undertaken by the university between January 2018 and August 2019, were included. The participants (employees of TUMS) were interviewed at the TEC center of the university, and a questionnaire was used to collect data on their mental and physical health. Participants were included if they were employed by TUMS under any type of employment status during the study period and provided informed consent. Job title, employment status, and occupational group (including office and administrative services, clinical and medical services, public services, technical services, diagnostic and chemical laboratory staff, and security guards) were recorded for all participants (35).

### Data collection

Data related to the different aspects of SB were collected using the third version of the World Mental Health Composite International Diagnostic Interview (CIDI) (36). The CIDI assesses participants' lifetime occurrence, age of onset, and recency of suicidal ideation (“Have you ever seriously thought about attempting suicide?”), suicide planning (“Have you ever made a plan for attempting suicide?”), and suicide attempts (“Have you ever attempted suicide?”). The questionnaires were completed through interviews, which were conducted by trained personnel.

Independent variables assessed in this study included personal and social determinants of health. These were age, gender, marital status, educational level, childhood socioeconomic status (SES), current SES, degree of fluctuation in SES, occupational group, employment status, job position, number of people in the household, number of books read in the past year (except for textbooks, business books, religious books, and holy books), work experience, social capital, and household assets.

Household assets assessed included a car (not for commercial purposes), a dishwasher, a microwave oven, a personal computer, a washing machine, a color television, or a video system (VHS, VCD, or DVD), as well as Internet access at home. Number of rooms and per capita area of the building; frequency of concert, theater, and movie attendance; frequency of eating a restaurant meal with the cost covered by the respondent; frequency of taking a flight; and monthly Internet cost were combined using categorical principal components analysis (CATPCA). The SES of the participants was selected as the first factor, and this was divided into five categories (percentiles) (37, 38). The respondents were thus classified into five groups based on their SES category, from high SES (richest) to low SES (poorest).

### Data analysis

Descriptive analyses were conducted to assess the distribution of sociodemographic variables and SBs among the participants. Data on continuous variables are reported in the form of means and standard deviations, and categorical data are reported as frequencies and percentages. Differences in SBs among different subgroups were explored using Chi-square analysis.

Univariate and multivariate logistic regressions were performed to identify the risk factors for SBs. Each independent variable was first entered separately into a univariate logistic regression analysis, and variables displaying an association with *p* < 0.2 were entered into a multiple logistic regression model. Crude and adjusted odds ratios (ORs) with their corresponding 95% confidence interval (95% CI) were recorded. All statistical analyses were conducted using SPSS 24. The threshold for statistical significance was defined as a *p*-value < 0.05.

## Results

Among the 3,913 participants enrolled in this study, 2,371 (60.6%) were women, and 3,166 (80.9%) were married. The mean age of the participants was 41.73 years (SD: 8.83, range: 20–75). Overall, 49.6% of respondents (*n* = 1,939) reported that they were sometimes tired of life and thought about death. The lifetime prevalence rates of SI (*n* = 317), SP (*n* = 287), and SA (*n* = 122) were 8.1%, 7.3%, and 3.1%, respectively. Table 1 presents the incidence of suicidal behaviors among different sociodemographic groups.

**Table 1:** The distribution of being tired of life and thinking about death and of suicidal behaviors according to participant characteristics.

**Variable**	**Sample *N* (%)**	**Tired of life and thinking about death**	**SI**	**SP**	**SA**
Total	3,913 (100)	1,939 (49.6)	317 (8.1)	287 (7.3)	122 (3.1)
**Gender**					
Male	1,542 (39.4)	620 (40.2)	89 (5.8)	84 (5.4)	33 (2.1)
Female	2,371 (60.6)	1,319 (55.6)	228 (9.6)	203 (8.6)	89 (3.8)
**Age**					
20–30	385 (9.8)	221 (57.4)	43 (11.2)	39 (10.1)	13 (3.4)
31–40	1,492 (38.1)	785 (52.6)	131 (8.8)	109 (7.3)	47 (3.2)
41–50	1,334 (34.1)	645 (48.4)	104 (7.8)	99 (7.4)	44 (3.3)
Over 50	702 (17.9)	288 (41)	39 (5.6)	40 (5.7)	18 (2.6)
**Marital status**					
Never married	628 (16)	318 (50.6)	61 (9.7)	49 (7.8)	17 (2.7)
Married	3,166 (80.9)	1,563 (48.5)	237 (7.5)	221 (7)	93 (2.9)
Divorced	79 (2)	59 (74.7)	15 (19)	13 (16.5)	11 (13.9)
Widowed	40 (1)	26 (65)	40 (10)	4 (10)	1 (2.5)
**Education level**					
Primary and middle school	342 (8.7)	173 (50.6)	34 (9.9)	35 (10.2)	18 (5.3)
Diploma	712 (18.2)	371 (52.1)	74 (10.4)	72 (10.1)	34 (4.8)
Bachelor's degree	1,818 (46.5)	909 (50)	142 (7.8)	125 (6.9)	46 (2.5)
Master's degree/GP	831 (21.2)	400 (48.1)	56 (6.7)	45 (5.4)	21(2.5)
MD specialist/Ph.D.	210 (5.4)	86 (41)	11 (5.2)	10 (4.8)	3 (1.4)
**Household wealth quintile**					
Richest	757 (19.3)	385 (50.9)	73 (9.6)	69 (9.1)	34 (4.5)
Rich	732 (18.7)	372 (50.8)	52 (7.1)	44 (6)	15 (2)
Intermediate	883 (22.6)	432 (48.9)	67 (7.6)	52 (5.9)	23 (2.6)
Poor	809 (20.7)	397(49.1)	70 (8.7)	68 (8.4)	28 (3.5)
Poorest	732 (18.7)	353 (48.2)	55 (7.5)	54 (7.4)	22 (3)
**Childhood socioeconomic status**					
High	172 (4.4)	79 (45.9)	12 (7)	11 (6.4)	6 (3.5)
Moderate to high	654 (16.7)	345 (52.8)	57 (8.7)	47 (7.2)	23 (3.5)
Moderate	1,957 (50)	958 (49)	160 (8.2)	147 (7.5)	66 (3.4)
Moderate to low	604 (15.4)	309 (51.2)	50 (8.3)	44 (7.3)	16 (2.6)
Low	526 (13.4)	248 (47.1)	38 (7.2)	38 (7.2)	11 (2.1)
**Current socioeconomic status**					
High	184 (4.7)	89 (48.4)	12 (6.5)	11 (6)	3 (1.6)
Moderate to high	1,024 (26.2)	522 (51)	86 (8.4)	77 (7.5)	31 (3)
Moderate	2,118 (54.1)	1,048 (49.5)	170 (8)	158 (7.5)	75 (3.5)
Moderate to low	444 (11.3)	213 (48)	35 (7.9)	28 (6.3)	8 (1.8)
Low	143 (3.7)	67 (46.9)	14 (9.8)	13 (9.1)	5 (3.5)
**Fluctuation in socioeconomic status**					
Very much	154 (3.9)	69 (44.8)	12 (7.8)	9 (5.8)	4 (2.6)
Much	524 (13.4)	262 (50)	43 (8.2)	42 (8)	15 (2.9)
Neither little nor much	920 (23.5)	463 (50.3)	80 (8.7)	71 (7.7)	33 (3.5)
Little	754 (19.3)	371 (49.2)	62 (8.2)	58 (7.7)	27 (3.9)
Very little	1,561 (39.9)	774 (49.6)	120 (7.7)	107 (6.9)	43 (2.8)
**Occupational group**					
Administration	1,832 (46.8)	865 (47.2)	125 (6.8)	106 (5.8)	50 (2.7)
Clinical and medical services	1,530 (39.1)	808 (52.8)	147 (9.6)	131 (8.6)	51 (3.3)
Public services	551 (14.1)	266 (48.3)	45 (8.2)	50 (9.1)	21 (3.8)
**Employment status**					
Permanent position	1,608 (41.1)	755 (47)	132 (8.2)	123 (7.6)	56 (3.5)
Temporary position (on contract)	2,305 (58.9)	1,184 (51.4)	185 (8)	164 (7.1)	66 (2.9)
**Job position**					
Senior manager	56 (1.4)	29 (51.8)	3 (5.4)	2 (3.6)	1 (1.8)
Junior manager	142 (3.6)	67 (47.2)	12 (8.5)	11 (7.7)	7 (4.9)
Staff	2,355 (60.2)	1,171 (49.7)	186 (7.9)	166 (7)	65 (2.8)
Other	1,360 (34.8)	672 (49.4)	116 (8.5)	108 (7.9)	49 (3.6)
**Number of people in household**					
One	135 (3.5)	62 (45.9)	9 (6.7)	6 (4.4)	5 (3.7)
Two	751 (19.2)	391 (52.1)	62 (8.3)	59 (7.9)	22 (2.9)
Three	1,290 (33)	645 (50)	103 (8)	97 (7.5)	40 (3.1)
Four or more	1,737 (44.4)	841 (48.4)	143 (8.2)	125 (7.2)	55 (3.2)
**Book reading in the past year**					
No reading	1,647 (42.1)	827 (50.2)	136 (8.3)	127 (7.7)	51 (3.1)
One to two books	929 (23.7)	454 (48.9)	76 (8.2)	65 (7)	28 (3)
More than two books	1,337 (34.2)	658 (49.2)	105 (7.9)	95 (7.1)	43 (3.2)

Crude and adjusted odds ratios calculated to explore the associations of suicidal feelings, thoughts, and behaviors with various sociodemographic characteristics are shown in Tables 2, 3. In the crude model, female gender was associated with being tired of life and thinking about death [crude odds ratio (cOR) = 1.87, 95% CI: 1.64–2.12], as well as with all three types of SB: SI (cOR = 1.74, 95% CI: 1.35–2.24), SP (cOR = 1.62, 95% CI: 1.25–2.11), and SA (cOR = 1.78, 95% CI: 1.19–2.67). In the crude model, the odds of being tired of life and thinking about death, as well as the odds of experiencing SI and SP, decreased with increasing age. However, SA had no significant relationship with age in the crude model.

**Table 2:** Logistic regression models for being tired of life and thinking about death and for suicidal ideation according to sociodemographic characteristics.

**Characteristics**	**Tired of life and thinking about death**						**Suicidal ideation**					
	**Crude OR**			**Adjusted OR** ^*^			**Crude OR**			**Adjusted OR** ^*^		
	**OR**	**95% CI**	***p*-value**	**OR**	**95% CI**	***p*-value**	**OR**	**95% CI**	***p*-value**	**OR**	**95% CI**	***p*-value**
**Gender**												
Male (ref)	1						1					
Female	1.87	1.64–2.12	< 0.001				1.74	1.35–2.24	< 0.001			
**Marital status**												
Married (ref)	1						1					
Never married	1.09	0.92–1.29	0.33	0.83	0.69–0.99	0.04	1.33	0.99–1.79	0.05	1.06	0.78–1.45	0.70
Divorced	3.13	1.88–5.22	< 0.001	2.67	1.59–4.48	< 0.001	2.90	1.63–5.16	< 0.001	2.64	1.47–4.75	0.001
Widowed	1.97	1.03–3.79	0.04	2.01	1.04–3.91	0.04	1.37	0.49–3.89	0.55	1.52	0.53–4.38	0.44
**Education level**												
MD specialist/Ph.D. (ref)	1						1					
Master's degree/GP	1.34	0.99–1.82	0.06	1.06	0.77–1.45	0.73	1.30	0.67–2.54	0.43	1.02	0.52–2.00	0.96
Bachelor's degree	1.44	1.08–1.93	0.01	1.17	0.87–1.58	0.31	1.53	0.81–2.88	0.19	1.23	0.65–2.32	0.53
Diploma	1.57	1.15–2.14	0.005	1.54	1.12–2.13	0.01	2.09	1.09–4.03	0.03	2.01	1.04–3.89	0.04
Primary and middle school	1.48	1.04–2.09	0.03	1.69	1.18–2.41	< 0.001	2.06	1.02–4.16	0.04	2.28	1.12–4.63	0.02
**Household wealth quintile**												
Richest (ref)	1						1					
Rich	0.99	0.82–1.22	0.98	0.94	0.76–1.15	0.53	0.72	0.49–1.04	0.08	0.68	0.47–0.98	0.04
Intermediate	0.93	0.76–1.12	0.43	0.89	0.73–1.09	0.25	0.77	0.54–1.09	0.14	0.74	0.52–1.05	0.09
Poor	0.93	0.76–1.40	0.48	0.91	0.75–1.12	0.37	0.89	0.63–1.25	0.50	0.87	0.62–1.23	0.44
Poorest	0.90	0.73–1.10	0.30	0.87	0.70–1.06	0.16	0.76	0.53–1.10	0.14	0.74	0.51–1.06	0.10
**Childhood socioeconomic status**												
High (ref)	1						1					
Moderate to high	1.31	0.94–1.84	0.11	1.26	0.90–1.78	0.19	1.27	0.67–2.43	0.46	1.22	0.64–2.34	0.54
Moderate	1.13	0.83–1.54	0.45	1.07	0.78–1.47	0.67	1.19	0.65–2.18	0.59	1.14	0.62–2.10	0.68
Moderate to low	1.23	0.88–1.73	0.22	1.18	0.84–1.67	0.35	1.20	0.63–2.32	0.58	1.16	0.60–2.23	0.66
Low	1.05	0.74–1.48	0.78	1.03	0.72–1.46	0.89	1.04	0.53–2.04	0.91	1.01	0.52–1.99	0.97
**Current socioeconomic status**												
High (ref)	1						1					
Moderate to high	1.11	0.81–1.51	0.52	1.08	0.78–1.48	0.65	1.31	0.70–2.46	0.39	1.27	0.68–2.38	0.45
Moderate	1.05	0.77–1.41	0.77	1.01	0.74–1.37	0.96	1.25	0.68–2.29	0.47	1.21	0.66–2.23	0.53
Moderate to low	0.98	0.70–1.39	0.93	0.96	0.68–1.37	0.83	1.23	0.62–2.42	0.56	1.20	0.61–2.37	0.60
Low	0.94	0.61–1.46	0.79	0.97	0.62–1.51	0.89	1.56	0.70–3.48	0.28	1.60	0.71–3.58	0.26
**Fluctuation in socioeconomic status**												
Very little (ref)	1						1					
Little	0.99	0.83–1.17	0.86	0.97	0.82–1.16	0.77	1.08	0.78–1.49	0.65	1.07	0.78–1.47	0.68
Neither little nor much	1.03	0.88–1.21	0.72	1.03	0.87–1.21	0.76	1.44	0.86–1.54	0.37	1.14	0.85–1.54	0.37
Much	1.02	0.83–1.24	0.86	1.00	0.82–1.23	0.98	1.07	0.75–1.54	0.70	1.06	0.74–1.52	0.76
Very much	0.83	0.59–1.15	0.25	0.85	0.60–1.19	0.34	1.02	0.55–1.88	0.96	1.04	0.56–1.94	0.90
**Occupational group**												
Administration (ref)	1						1					
Clinical and medical services	1.25	1.09–1.43	< 0.001	1.22	1.06–1.40	0.01	1.45	1.13–1.86	< 0.001	1.41	1.10–1.81	0.01
Public services	1.04	0.86–1.26	0.66	1.03	0.85–1.25	0.78	1.21	0.85–1.73	0.28	1.20	0.84–1.71	0.32
Employment status												
Permanent position (ref)	1						1					
Temporary position (on contract)	1.93	1.05–1.36	0.01	1.18	1.03–1.34	0.01	0.98	0.77–1.23	0.84	0.94	0.74–1.19	0.62
**Job position**												
Senior manager (ref)	1						1					
Junior manager	0.83	0.45–1.55	0.56	0.84	0.45–1.58	0.59	1.63	0.44–6.01	0.46	1.67	0.45–6.17	0.44
Staff	0.92	0.54–1.57	0.76	0.89	0.52–1.53	0.68	1.52	0.47–4.90	0.49	1.47	0.45–4.75	0.52
Others	0.91	0.53–1.55	0.73	0.89	0.52–1.53	0.67	1.65	0.50–5.35	0.40	1.60	0.49–5.21	0.44
Age	0.98	0.97–0.98	< 0.001				0.98	0.96–0.99	< 0.001			
Work experience	0.99	0.98–0.99	0.01	0.99	0.99–1.00	0.04	1.04	0.99–1.02	0.46	1.01	0.99–1.02	0.34
Social capital	0.99	0.99–1.01	0.07	0.99	0.99–1.00	0.07	0.99	0.98–1.01	0.83	0.99	0.99–1.01	0.82
Number of people in household	0.96	0.91–1.02	0.17	0.96	0.91–1.02	0.22	1.03	0.93–1.14	0.64	1.03	0.93–1.14	0.57
Book reading in the past year	1.06	0.98–1.03	0.64	0.99	0.98–1.01	0.64	0.98	0.94–1.03	0.51	0.98	0.96–1.01	0.26

^*^Adjusted for sex and age.

**Table 3:** Logistic regression models for suicide planning and suicide attempts according to sociodemographic characteristics.

**Characteristics**	**Suicide planning**						**Suicide attempts**					
	**Crude OR**			**Adjusted OR** ^*^			**Crude OR**			**Adjusted OR** ^*^		
	**OR**	**95% CI**	***p*-value**	**OR**	**95% CI**	***p*-value**	**OR**	**95% CI**	***p*-value**	**OR**	**95% CI**	***p*-value**
**Gender**												
Male (ref)	1						1					
Female	1.62	1.25–2.11	< 0.001				1.78	1.19–2.67	0.01			
**Marital status**												
Married (ref)	1						1					
Never married	1.12	0.81–1.55	0.46	0.95	0.68–1.32	0.76	0.91	0.54–1.55	0.75	0.78	0.46–1.34	0.38
Divorced	2.62	1.42–4.83	< 0.001	2.35	1.27–4.36	0.01	5.34	2.73–10.4	< 0.001	4.61	2.33–9.12	< 0.001
Widowed	1.48	0.52–4.19	0.46	1.48	0.51–4.28	0.47	0.84	0.11–6.23	0.87	0.77	0.10–5.78	0.80
**Education level**												
MD specialist/Ph.D. (ref)	1						1					
Master's degree/GP	1.14	0.57–2.31	0.71	0.94	0.46–1.92	0.87	1.78	0.53–6.06	0.35	1.51	0.44–5.17	0.51
Bachelor's degree	1.48	0.76–2.86	0.24	1.25	0.64–2.44	0.51	1.79	0.55–5.81	0.33	1.57	0.48–5.14	0.46
Diploma	2.25	1.14–4.44	0.02	2.24	1.13–4.46	0.02	3.46	1.05–11.3	0.04	3.62	1.09–11.98	0.04
Primary and middle school	2.28	1.10–4.71	0.03	2.57	1.24–5.33	0.01	3.83	1.11–13.1	0.03	4.40	1.28–15.19	0.02
**Household wealth quintile**												
Richest (ref)	1						1					
Rich	0.63	0.43–0.94	0.02	0.61	0.41–0.90	0.01	0.44	0.24–0.82	0.01	0.42	0.23–0.78	0.01
Intermediate	0.62	0.42–0.90	0.01	0.61	0.42–0.88	0.01	0.56	0.33–0.97	0.04	0.55	0.32–0.95	0.03
Poor	0.91	0.64–1.29	0.62	0.90	0.63–1.28	0.56	0.76	0.45–1.27	0.29	0.75	0.45–1.25	0.27
Poorest	0.79	0.58–1.15	0.22	0.77	0.53–1.12	0.17	0.65	0.38–1.13	0.13	0.64	0.37–1.10	0.11
**Childhood socioeconomic status**												
High (ref)	1						1					
Moderate to high	1.13	0.57–2.23	0.71	1.09	0.55–2.15	0.81	1.08	0.40–2.51	0.98	0.96	0.38–2.39	0.92
Moderate	1.18	0.63–2.24	0.59	1.14	0.60–2.15	0.69	0.96	0.41–2.26	0.93	0.91	0.39–2.14	0.83
Moderate to low	1.15	0.58–2.27	0.68	1.11	0.56–2.20	0.77	0.75	0.29–1.95	0.56	0.72	0.28–1.86	0.49
Low	1.14	0.56–2.28	0.71	1.11	0.56–2.23	0.76	0.59	0.21–1.62	0.30	0.57	0.21–1.58	0.28
**Current socioeconomic status**												
High (ref)	1						1					
Moderate to high	1.27	0.66–2.45	0.46	1.24	0.65–2.38	0.52	1.88	0.57–6.22	0.29	1.81	0.55–5.99	0.33
Moderate	1.26	0.67–2.38	0.46	1.23	0.65–2.31	0.52	2.21	0.69–7.09	0.18	2.13	0.66–6.83	0.20
Moderate to low	1.05	0.51–2.17	0.87	1.04	0.50–2.13	0.92	1.10	0.29–4.22	0.88	1.08	0.28–4.12	0.91
Low	1.57	0.68–3.62	0.28	1.60	0.70–3.70	0.27	2.18	0.51–9.30	0.29	2.23	0.52–9.48	0.28
**Fluctuation in socioeconomic status**												
Very little (ref)	1						1					
Little	1.13	0.81–1.57	0.46	1.12	0.81–1.57	0.50	1.31	0.80–2.13	0.27	1.29	0.79–2.11	0.31
Neither little nor much	1.13	0.83–1.55	0.42	1.13	0.83–1.55	0.44	1.31	0.82–2.08	0.24	1.30	0.82–2.07	0.26
Much	1.18	0.81–1.71	0.37	1.18	0.81–1.71	0.39	1.04	0.57–1.88	0.89	1.04	0.57–1.89	0.89
Very much	0.84	0.41–1.70	0.63	0.86	0.43–1.74	0.68	0.94	0.33–2.65	0.90	0.97	0.34–2.73	0.95
**Occupational group**												
Administration (Ref)	1						1					
Clinical and medical services	1.52	1.70–1.99	< 0.001	1.50	1.15–1.96	< 0.001	1.23	0.82–1.83	0.31	1.22	0.82–1.82	0.32
Public services	1.62	1.14–2.30	0.01	1.61	1.13–2.29	0.01	1.41	0.84–2.37	0.19	1.40	0.83–2.35	0.21
**Employment status**												
Permanent position (ref)	1						1					
Temporary position (on contract)	0.92	0.72–1.17	0.52	0.91	0.71–1.17	0.48	0.81	0.56–1.17	0.27	0.83	0.57–1.19	0.31
**Job position**												
Senior manager (ref)	1						1					
Junior manager	2.26	0.48–10.5	0.29	2.29	0.49–10.69	0.29	2.85	0.34–23.7	0.33	2.84	0.34–23.66	0.34
Staff	2.04	0.49–8.47	0.32	2.01	0.48–8.31	0.34	1.56	0.21–11.4	0.66	1.53	0.21–11.27	0.67
Others	2.23	0.56–9.64	0.24	2.30	0.55–9.56	0.25	2.05	0.27–15.1	0.48	2.05	0.28–15.12	0.48
Age	0.99	0.97–0.99	0.03				0.99	0.97–1.01	0.51			
Work experience	1.08	0.99–1.02	0.16	1.01	0.99–1.02	0.13	1.01	0.99–1.01	0.15	1.01	0.99–1.03	0.15
Social capital	0.99	0.98–1.05	0.29	0.99	0.98–1.01	0.30	0.99	0.98–1.01	0.66	0.99	0.98–1.01	0.68
Number of people in household	1.01	0.90–1.12	0.82	1.02	0.91–1.13	0.78	1.03	0.88–1.21	0.63	1.04	0.89–1.22	0.62
Book reading in the past year	0.98	0.93–1.04	0.54	0.99	0.96–1.02	0.37	0.98	0.91–1.06	0.67	0.99	0.96–1.04	0.84

^*^Adjusted for sex and age.

Compared with married employees, divorced employees were much more likely to report feeling tired of life and thinking about death [adjusted odds ratio (aOR) = 2.67, 95% CI: 1.59–4.48], and to report having engaged in all three types of suicidal behavior (SI: aOR = 2.64, 95% CI 1.47–4.75; SP: aOR = 2.35, 95% CI 1.27–4.36; and SA: aOR = 4.61, 95% CI 2.33–9.12). In terms of employment group, compared with the administrative services group, people working in clinical and medical services and those directly involved in providing medical and therapeutic services to patients were at a higher risk of SI (aOR = 1.41, 95% CI: 1.10–1.81) and SP (aOR = 1.50, 95%CI: 1.15–1.96), as well as a higher risk of feeling tired of life and thinking about death (aOR = 1.22, 95% CI: 1.06–1.40). In the crude and adjusted analyses, respondents with education levels no higher than a diploma had significantly increased odds of reporting being tired of life and thinking about death, and of reporting suicidal behaviors. Employment status was only significantly associated with feeling tired of life and thinking about death. Compared with permanent employees, employees working under temporary contracts had increased odds of reporting feeling tired of life and thinking about death (aOR = −1.18, 95% CI: 1.03–1.34). Generally, in both the crude and the adjusted model, household wealth quintile was significantly associated with risk of SP and SI. In comparison with the richest quintile, the odds of SP in the rich and intermediate-wealth quintiles were 39% lower. In addition, the odds of SA in the rich and intermediate-wealth quintiles were 58 and 45% lower, respectively.

## Discussion

Employee mental health is a key resource for good quality of life, wellbeing, and work. Therefore, monitoring employee mental health should be an element of workplace health promotion and development plans and programs. This study showed that approximately half of the employees studied reported that they felt tired of life and had thought about death.

These characteristics may fall within the concept of “demoralization.” Demoralization is experienced as existential despair, hopelessness, helplessness, and loss of meaning and purpose in life. Hopelessness, the hallmark of demoralization, is associated with poor outcomes in physical and psychiatric illness and, importantly, with SI and the wish to die (39). A previous systematic review showed that demoralization could be associated with SI/SB and with a significant increase in suicide risk (40). The results of this study showed that almost 10% of healthcare professionals reported having experienced SI (8.1%), SP (7.3%), or SA (3.1%). According to a WHO report, Iran's crude suicide rate for all ages (per 100,000) in 2019 was 5.2 (41). A study conducted in the USA showed that 5% of suicide deaths from 2003 to 2016 occurred among healthcare professionals (42).

Similar to other studies, this study showed that thinking about death, SI, SP, and SA were more prevalent in women than in men (but less than twice as prevalent) (43, 44). A previous systematic review and meta-analysis indicated that women were at approximately twice as great a risk of SA and men approximately three times as great a risk of suicide death (45). In addition, similar to previous studies (including a European cross-national study of 5,212 participants), this study showed that SAs by men appeared to be more serious than SAs by women (46–51). Men are less likely to seek social support, indicating that the goal of suicide attempts by men is more likely to be to die rather than to seek help (52–54). Since patterns of suicidal behavior differ by gender, these differences should be considered carefully in the design of preventive intervention programs to address suicide.

The data analysis also showed that an increase in age protects individuals against feeling tired of life, thinking about death, and SI. A similar effect has been reported in a nationally representative survey conducted in the USA, which reported that individuals younger than 26 years were more likely to report having major depressive disorder and suicidal thoughts, and were more likely to attempt suicide and/or to die by suicide as compared to adults aged 26 years or older (51). These results strongly indicate that adolescents are a high-risk group for suicide-related behaviors.

Marital status was also found to influence the risk of suicide in this study, as divorced employees were more likely to feel tired of life, think about death, and engage in each of the three types of suicidal behavior (SI, SP, and SA). Similar associations between marital status and suicide have been reported in other studies (52, 53): specifically, divorcees are more likely to report having thoughts of “life not worth living,” major depression, panic and anxiety disorders, and low self-esteem, which are known risk factors for suicide (53). However, there are also contradictory findings. For example, one study in the USA showed that healthcare professionals who were married were at a higher risk of suicide than those who were not married (42). This is probably because of issues related to the quality of married life, as observed in a study conducted in China, where a significant relationship with suicide or SA was found for marital conflicts and quarreling with a partner within the past month (54).

Studies that have measured rates of suicide in different occupational groups have shown that suicide rates vary across these groups (55, 56). The present study also showed that type of job influences risk of suicide. People working in clinical and medical services were more likely to be at risk for suicidal behaviors than those working in administration. A study conducted in the United States showed that the suicide rate of medical professionals is noticeably higher than that of the general population. For example, the risk of dying by suicide is three times higher for surgeons than it is for the general population (57). Another study in Denmark reported that medicine and nursing are the occupations with the highest suicide rate (58). The effect of job type on suicide may be due to high levels of stress in some occupations (59). Furthermore, burnout is also a risk factor in healthcare professions (60), and this is reportedly associated with SI (61). Other potential reasons might be that health professionals are more aware of suicide methods (for example, different kinds of drugs, lethal doses, and their effects) and have better access to these methods (62).

This study also indicated that lower income, lower education levels, and lower occupation levels might be risk factors for feeling tired of life and thinking about death, and for suicidal behaviors. A study conducted among Korean employees showed that workers with fewer years of education experienced more SI (63). Similar to certain other studies (64), the present study identified a significant association between employment status and having a sense of being tired of life and thinking about death. Socioeconomic factors, such as level of education, income, and employment, are interrelated variables, the most important of which can be level of education. People with higher levels of education are more likely to have better jobs, and consequently, they are more likely to have higher incomes. Exposure to poor working conditions is associated with poor mental health, which can be a predictor of suicide (33). Therefore, level of education can function as a fundamental factor in suicide. According to the findings, the top priority groups for suicide prevention programs in the workplaces studied should be women, especially divorced women with low levels of education.

In conclusion, this study has identified social determinants of employees' mental health, such as being female, being divorced, and having a low level of education, which were found to significantly affect both thoughts and behaviors even after adjusting for socioeconomic variables. The results also confirmed that most suicide risk factors present in the general population also applied to the employees of the medical university studied. This finding highlights the need to prioritize mental health and to focus on high-risk groups (women, divorced individuals, and those with a low level of education) for suicide prevention in workplace programs and policies promoting mental health, as well as in research and clinical practice. For example, divorced female employees with limited education should be considered a priority group to utilize mental health consultation and treatment provided by mental health practitioners and clinicians.

## Strengths and limitations

The study partially reported in this article is a major workplace health cohort project with a relatively large and highly diverse representative sample consisting of individuals from different socioeconomic backgrounds. Another notable strength of the study is the investigation of suicide-related feelings, in addition to SI, SP, and SA. However, the findings of this cross-sectional study are limited in terms of the possibility of making any causal interpretation. Therefore, the results must be interpreted with caution.

## Data availability statement

The raw data supporting the conclusions of this article will be made available by the authors, without undue reservation.

## Ethics statement

The studies involving human participants were reviewed and approved by Tehran University of Medical Sciences. The patients/participants provided their written informed consent to participate in this study.

## Author contributions

ZR, AA, NK-M, and AL contributed to the study conception and design, drafting of the manuscript, and critical revision of the manuscript for important intellectual content. SM and HP participated in the acquisition of data, drafting of the manuscript, and critical revision of the manuscript for important intellectual content. All authors agreed on the final manuscript prior to submission and agreed to be accountable for all aspects of this work.
